# The Green Cochlea

**DOI:** 10.1016/j.bjorl.2025.101718

**Published:** 2025-09-06

**Authors:** Abdulrahman Hagr, Farid Alzhrani, Fida Almuhawas, Yassin Abdelsamad, Medhat Yousef, Asma Alahmadi, Christiana Kyvelidou, Ilona Anderson

**Affiliations:** a King Abdullah Ear Specialist Center (KAESC), King Saud University Medical City, King Saud University, Riyadh, Saudi Arabia; b Research Department, MED-EL GmbH, Riyadh, Saudi Arabia; c Clinical Research Department, MED-EL GmbH, Innsbruck, Austria

**Keywords:** Cochlear implantation, Green cochlea, Sustainability, Anesthesia, Sustainable Development Goals (SDGs), Early Activation

## Abstract

**Objectives:**

Healthcare systems contribute significantly to global greenhouse gas emissions through energy consumption and waste generation. This study aims to explore strategies to make cochlear implantation processes more environmentally sustainable and aligned with the United Nations’ Sustainable Development Goals.

**Methods:**

We examined various approaches including the use of bio-based and biodegradable materials, sustainable energy solutions, greener anesthetic practices, effective waste separation and recycling in operating rooms, and patient-centered strategies such as reducing travel and promoting early activation and fitting of cochlear implants.

**Results:**

Establishing a ‘Green Team’ was identified as a key factor to raise awareness and motivation for sustainability initiatives. Implementing these strategies can significantly reduce the environmental footprint of cochlear implantation by decreasing surgical waste, lowering energy consumption, and minimizing patient-related emissions.

**Conclusion:**

By adopting these sustainable practices, the cochlear implant field can reduce its environmental impact, contribute meaningfully to global sustainability efforts, and enhance patient well-being, positioning itself as a leader in healthcare sustainability.

## Highlights

• Cochlear implants can adopt greener materials and sustainable practices.
• Green Teams drive awareness and action in cochlear implant sustainability.
• Early activation reduced travel lower patient-related emissions.
• Greener anesthesia and OR recycling cut the surgical environmental impact.
• The cochlear implant field can lead in aligning healthcare with SDGs.

## Introduction

Healthcare systems contribute 4%–5% of global greenhouse gas emissions. Of these, 15%–50% are direct emissions from energy use or indirect emissions from purchased electricity, while 50%–75% stem from indirect emissions which are primarily associated with disposables, equipment (both medical and non-medical), and pharmaceuticals.^1^ Sustainability in medical practices is increasingly recognized as essential for developing an environmentally responsible and economically feasible healthcare system.

**Fig. 1:**
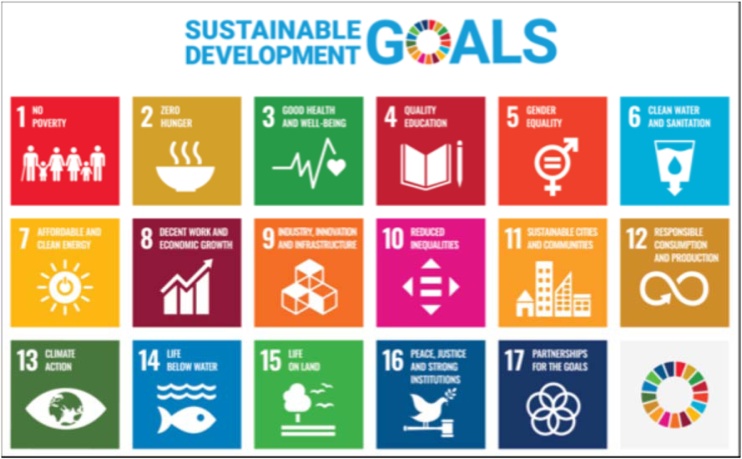
Overview of United Nations’ Sustainable Development Goals (SDGs).

The Environmental, Social, and Governance (ESG) criteria and the United Nations’ Sustainable Development Goals (SDGs) are broader frameworks which provide structured approaches for incorporating more sustainable practices.^2^ ESG criteria emphasize operational practices that are environmentally sound, socially responsible, and governed through ethical policies. The SDGs are 17 interlinked global objectives aimed at achieving a better and more sustainable future for all people worldwide and for the benefit of present and future generations (Fig. 1). They were set in 2015 by the United Nations General Assembly and are intended to be achieved by the year 2030. SDGs relevant to healthcare include good health and well-being (goal 3), responsible consumption and production (goal 12), and climate action (goal 13).

As the healthcare sector works towards reducing its environmental impact and transition to net-zero emissions, the Cochlear Implant (CI) field has an opportunity to lead the way in adopting more sustainable practices. CIs are devices that restore hearing to people with severe to profound hearing loss, substantially improving their quality of life and overall well-being.^3^

This article aims to identify specific areas within the CI field where sustainable practices can and should be adopted to establish the ‘Green cochlea’,^4^ a sustainable CI approach. It also connects each innovating action with United Nation’s SDGs.

### Sustainability in healthcare

Traditional healthcare practices can generate substantial waste, contribute to pollution, and consume large amounts of energy and resources. Emergencies and crises can further escalate the use of disposable items, as seen during the COVID-19 pandemic, when nearly 129 billion face masks and 65 billion gloves were discarded worldwide each month.^5^ Sustainable healthcare seeks to minimize these negative impacts while promoting patient well-being, reducing costs, and improving efficiency.

To move toward a more sustainable healthcare, it is essential to evaluate the areas contributing most to the environmental footprint. Operating Rooms (ORs) have been identified as major contributors to energy consumption and waste generation.^6^ A recent study found that although doctors in ORs scored the highest in sustainability knowledge, they were significantly less likely to practice sustainable measures versus non-doctors, and most respondents were unwilling to adopt any practice changes.^6^ Anesthesia is another major contributor, a prospective audit done at the Western Hospital in Australia found that one quarter of the general waste (classified as non-infectious waste) comes from anesthesia, with 58% of this waste being recyclable.^7^ Despite interest in recycling, it is estimated that less than one third of anesthesiologists actively did so in the OR.^8^ A recent qualitative study using the Behavior Change Wheel framework explored anesthesiologists' perceptions and adoption of environmentally sustainable practices, identifying areas such as recycling bin availability, reduction of single-use items, and usage of agents with a lower carbon footprint as key areas of improvement. In addition, training on green practices and the involvement of senior anesthesiologists as role models were identified as areas to be strategically addressed.^9^

### Overview of cochlear implantation

CIs are devices that restore hearing to people with congenital or acquired severe to profound sensorineural hearing loss. The CI system comprises both external and internal components. External components include an audio processor which houses a microphone, signal processing circuitry, and a transmitting coil. Internal components consist of the implant and the electrode array. The microphone captures sound from the environment which is processed into digital signals. These are transmitted into the internal implant and converted into electric pulses, which are delivered to the auditory nerve via an intracochlear electrode array, bypassing the non-functioning parts of the ear. Different electrode contacts on the array are activated based on the frequency of the sound signals and can stimulate different parts of the cochlea. Finally, the auditory nerve transmits these electrical impulses to the brain, where they are interpreted as sound.

Prior to implantation, candidates for CI go through a series of hearing assessments to determine the degree of their hearing loss. During this time, they require multiple appointments with various healthcare professionals. An ENT surgeon is responsible for the medical evaluation, diagnosis, surgery, and the medical follow-up. An audiologist is responsible for the evaluation of hearing function, programming of the CI and the audiological follow-up. A rehabilitationist or speech therapist is responsible for the evaluation of speech and language progress and the rehabilitation. In addition, imaging tests, such as Computed Tomography (CT) scans or Magnetic Resonance Imaging (MRI) might be required pre- or postoperatively.

For the standard cochlear implantation procedure, the patient is placed under general anesthesia. An incision is made behind the ear (postauricular) to access the mastoid bone. A small cavity is drilled in the mastoid bone (mastoidectomy), followed by a small opening from the mastoid to the middle ear (posterior tympanotomy) in order to expose the round window of the cochlea. The electrode array is inserted in the scala tympani, while the implant is positioned on the skull bone and securely fixed. The incision is closed with sutures, and a sterile dressing is applied. Overall, the average total operative room time is estimated to be approximately 2–3 hours; however, this can vary depending on several factors.^10^^,^^11^

In the immediate postoperative period, the patient may be administered pain relief medication and/or antibiotics to manage pain and prevent infection. CI activation takes place within 24 h to 6 weeks, when an audiologist activates and programs the CI, followed by fitting when the appropriate electrical stimulus levels for each electrode are set using either a standard fitting map or a personalized Anatomy-Based Fitting (ABF) map. Additional fitting sessions are scheduled to fine-tune the device based on the user’s feedback and progress, while auditory and speech training begin immediately to help the CI user adapt to the new sound input.

For people who need it, access to a CI ensures healthy living and promotes well-being for all at all ages (SDG3), lifelong learning opportunities (SDG4) and employment (SDG 8) for all, inclusivity and safety (SDG 11 and 16), and reduces inequality (SDG10). Moreover, access to a CI for people who need it defends patient safety rights according to the World Health Organization (WHO) by securing the right to timely, effective, safe, and appropriate care by qualified and competent safe workers in safe and secure health facilities.^12^

## Sustainable alternatives in CI materials

The material properties of traditional petroleum-derived plastics make them ideal for many applications, including medical devices and packaging. However, their widespread use has led to an accumulation of plastic waste and thus the healthcare sector should look towards eco-friendly and sustainable alternatives. Bio-based polymers (also known as bioplastics) and biodegradable plastics are promising alternatives.^13^ Although more research is needed to determine their efficacy for use in medical devices, they should be considered as greener alternatives for packaging material.

Apart from the plastic compartments, a substantial amount of paper is required for the mandatory printed manuals and safety instructions that accompany each implant in multiple languages. Both CI users and clinicians can advocate for regulatory changes that promote sustainability, such as digital manuals and instructions which can reduce paper waste. Until such regulatory changes are made, the use of recyclable non-illustrated paper should be prioritized whenever possible.^14^

CIs require high-power batteries which, as all batteries, have a large environmental impact.^15^ Traditional disposable batteries, such as zinc-air, silver-oxide, and alkaline batteries contribute to substantial waste and resource depletion. Transitioning to more sustainable battery solutions, such as rechargeable systems, can offer several environmental and economic benefits.

To accurately estimate the overall environmental impact of each device, a Life Cycle Assessment (LCA) is needed. According to the European Environment Agency, an LCA is “a process of evaluating the effects that a product has on the environment over the entire period of its life thereby increasing resource-use efficiency and decreasing liabilities”.^16^ Conducting an LCA will enable us to identify areas with the highest environmental impact and focus efforts to finding greener alternatives.

Creating a sustainable CI ensures sustainable consumption and production patterns (SDG12) and fosters innovation (SDG9). In addition, transitioning to more sustainable battery solutions promotes access to affordable, sustainable, and modern energy for all (SDG7).

## Sustainable surgical practices

### Choice of anesthesia

There are various anesthetic methods, each with a distinct impact on the environment. General anesthetic methods can be classified into inhaled or Intravenous (IV). Inhaled anesthetics can be further subdivided into two distinct classes: Nitrous Oxide (N_2_O) and volatile halogenated agents (e.g., isoflurane, desflurane, sevoflurane). N_2_O is supplied in a gas form, whereas halogenated agents are in liquid form and need to be vaporized by an anesthesia machine. N_2_O has several advantageous properties as an anesthetic and when combined with halogenated agents, it reduces their minimum alveolar concentration, thereby requiring a smaller amount of a halogenated agent to achieve the same level of anesthesia. Both volatile halogenated agents and N_2_O are potent greenhouse gases, contributing to global warming and potentially damaging earth’s ozone layer.^17^ The emissions of an anesthetist's daily routine, even when using low amounts gases, can be so high that it is comparable to driving over 1000 km every day. As a result, the American Society of Anesthesiologists recommends avoiding desflurane and N_2_O when possible, using low-flow anesthesia techniques, and considering alternatives like local anesthesia or Total Intravenous Anesthesia (TIVA) when appropriate.^18^

In a large-scale comparative study, the exclusive use of TIVA was found to drastically reduce the carbon footprint of hypnotic drugs in general anesthesia. The study demonstrated that the carbon dioxide equivalent footprint per intervention was 20 times lower for the TIVA strategy (2.42 kg) compared to a mixed TIVA-volatile strategy (48.85 kg) in adult patients.^19^

Propofol is a widely used IV anesthetic that has a lower environmental impact since, unlike inhaled anesthetics, it is not released into the air. However, it should be disposed via incineration due to its toxicity to aquatic organisms.^20^^,^^21^ Local anesthesia is a greener alternative which can reduce a procedure’s carbon footprint.^22^ Moreover, it has been successfully used in cochlear implantation in combination with sedation and can reduce postanesthesia side-effects like nausea and vomiting, shorten postoperative recovery time, and can be safer than general anesthesia for elderly people. Toner et al. were the first in 1998 to successfully perform a mastoidectomy followed by posterior tympanotomy in 4 patients under local anesthesia with sedation.^23^ Subsequent studies by Hamerschmidt et al. confirmed the safety and efficacy of local anesthesia.^24^^,^^25^ Aldhafeeri et al. reported the feasibility of performing cochlear implantation under conscious sedation with same-day fitting, showing the benefits and practicality of using local anesthesia in cochlear implant procedures, including reduced recovery time and the potential for patients to be discharged the same day. Their approach minimized complications associated with general anesthesia and allowed for early activation of the CI.^26^ More recently, in a retrospective case-cohort study, all 117 people undergoing cochlear implantation under local anesthesia with or without conscious sedation were successfully operated and 90% of them reported that the surgery was a positive experience.^27^ In a systematic review and meta-analysis, Walterns et al. found that surgical time was significantly shorter under local anesthesia, and only minor complications were reported among the 18 studies included.^28^ Similarly, a meta-analysis by Avallone et al. demonstrated that local anesthesia in CI surgery resulted in a shorter surgical time and lower costs compared to general anesthesia, without significantly affecting hospitalization or time in the postanesthesia care unit. In addition, they report that local anesthesia is gaining popularity, especially among elderly patients.^29^ Using local anesthetics in CI surgeries appears to be beneficial both to patients and healthcare professionals by shortening the procedure time and reducing exposure to more hazardous chemicals, while also being beneficial to the environment. Conducting an LCA would help determine with better accuracy the relative environmental footprint of local anesthesia compared to TIVA and inhaled anesthetics.

Apart from the choice of the anesthetic, minimizing drug waste is crucial. Drug waste can account for up to 26% of the total anesthesia drug budget, representing a substantial economic burden and a considerable environmental impact.^21^

Using local anesthesia promotes well-being for all ages (SDG3), ensures sustainable consumption patterns (SDG12), and by reducing the use of inhaled anesthetics, takes urgent action to combat climate change (SDG13). Addressing the drug waste problem ensures sustainable consumption patterns (SDG12). Reducing the postoperative time and the time spent in the clinic promotes well-being for all ages (SDG3).

### Minimization of surgical waste

Simple behavioral changes in the OR and effective waste separation could lead to substantial environmental and economic benefits. In a pilot study at the Department of General Surgery, University of Turin, Italy, a recycling program was implemented for 18 months in a single operating room and involved the separation collection of plastic, paper, non-woven fabric, and biohazardous waste.^30^ The creation of a multidisciplinary ‘Green Team’ which promoted the ‘5Rs’: Reduce, Reuse, Recycle, Rethink, and Research, was crucial to the project's success. The study reported a substantial reduction in CO_2_ emissions and cost savings due to decreased incineration of biohazardous waste. In addition, the study recorded a total of 1369 surgical procedures, categorized by complexity, and found that minor surgeries contributed significantly to biohazardous waste due to their frequency. Apart from recycling, employing reusable OR tools, and especially anesthetic tools (like supraglottic airways, laryngoscopes, direct-contact heaters, slide sheets, and drug trays), can lead to significant cost savings and reduce the OR carbon footprint by up to 84%.^31^ The same can apply not only to OR tools but textiles such as theatre hats, surgical gowns, and drapes, and even practices such as hand decontamination.^32^ Reducing waste in the OR room can be further achieved by avoiding the opening of equipment, especially single-use items, in anticipation of the surgeon’s needs, as many such supplies end up being disposed unused.^33^ Similar green practices can be easily adopted in CI surgeries by replacing single-use items where possible, placing clearly labeled bins for non-contaminated material and training the OR personnel on avoiding the unnecessary opening of equipment, and on proper waste separation and disposal.

Minimizing surgical waste ensures sustainable consumption and production patterns (SDG12) and combats climate change (SDG13). Creating and promoting a ‘Green Team’ can have various implications such as ensuring sustainable management of water (SDG6) and sustainable and modern energy (SDG7), promoting productive employment (SDG8), fostering innovation (SDG9), making cities sustainable (SDG11), ensuring sustainable consumption and production patterns (SDG12), building accountable and inclusive institutions (SDG16), and taking urgent action to combat climate change and its impacts (SDG13).

## Postimplantation processes

### Postimplantation care

Prophylactic use of antibiotics is a common practice in surgeries. However, as pharmaceuticals, antibiotics pose several ecological risks to soil microorganisms and aquatic life due to improper disposal methods during and after their production.^34^ Moreover, unnecessary antibiotic use is a major contributor to the alarming rise of antibiotic resistance, estimated to account for 1.27 million deaths globally per year.^35^

A more judicious use of antibiotics can promote well-being for all ages (SDG3), ensure sustainable consumption patterns (SDG12), and by minimizing their use and improper disposal, conserve the oceans (SDG14) and take urgent action to combat climate change (SDG13).

### Activation and fitting

The activation and subsequent fitting of a CI are critical stages in the postimplantation process. Activation involves turning on and programming the sound processor for the first time, marking the first time that the CI user will hear through the device. Fitting refers to the fine-tuning of the CI to optimize the auditory performance for the user. Traditionally, activation occurs 3–6 weeks postimplantation, a relatively long period that ensures adequate wound healing. However, early activation and fitting within 2 weeks postimplantation have been shown to be feasible and safe without any significant long-term effects regarding wound healing and speech recognition compared to the traditional activation and fitting.^36^ Going one step further, next-day and even same-day activation and fitting have also proven a feasible and safe, with early activation not interfering with wound healing or causing any serious complications. The shift from weeks to as early as a day for CI activation may enhance patient outcomes, operational efficiency, and impact the economic and rehabilitation aspects of cochlear implantation.^37^^,^^38^ A study investigating the long-term effects of early activation on electrode impedance over a period of two years in a cohort of 915 ears was the first to introduce the term ‘Green cochlea’.^4^ The study found that early activation led to a statistically significant and sustained reduction in the electrode impedance compared to traditional activation. Similar results were observed in pediatric CI users,^39^ suggesting that earlier activation might positively affect long-term CI outcomes. Reduced electrode impedance can also help increase the CI’s battery life, as higher electrode impedance depletes the device’s charge more quickly and shortens the batteries’ duration.^40^ Same-day activation is a viable option, and studies have shown that early next-day fitting can be routinely performed for CI patients.^26^^,^^41^ The manner of fitting is also important; better informed fitting with ABF takes into account each person’s unique cochlea anatomy and can increase speech performance and satisfaction in some CI users.^42^^,^^43^ Remote care offers a promising alternative to in-person fittings, proving to be as safe, feasible, and effective as local fitting for CI users of all ages.^44^ Moreover, some remote care applications, such as the HearCare application and MAESTRO 10 (MED-EL, Innsbruck, Austria), are compatible with ABF software, offering a tailored remote fitting for each CI user. Other applications, such as the Remote Check and Remote Assist (Cochlear, Sydney, Austria) or the Remote Programming through the AB Remote Support app (Advanced Bionics, Valencia, California, USA), can also offer remote care solutions to CI users.

Overall, performing cochlear implantation as day surgery is more effective for patients and providers, promotes well-being for all ages (SDG3), supports productive employment (SDG8), reduces travel time thus combating climate change (SDG13), and by possibly prolonging the CIs battery life, it impacts energy consumption (SDG7) and promotes responsible consumption and production (SDG12). Implementing patient-centered remote care and fitting promotes well-being for all ages (SDG3) and by reducing travel time, combat climate change (SDG13).

## Other innovations to sustainable practices

Multiple clinic visits can be time-consuming and inconvenient for patients. Combining multiple appointments into a single day can increase satisfaction.^45^ Furthermore, fewer visits can reduce travel time and decrease the carbon footprint associated with travel.^46^ To this end, various studies examined the efficacy and patient satisfaction of same-day consultation and implantation. Patro et al. conducted a study involving 35 adult CI candidates, with 9 successfully undergoing same-day consultation and implantation. The mean referral-to-surgery time was reduced from 103 to 52 days (adjusted for cancellations due to COVID-19) compared to traditional methods and participants rated their overall experience highly, with none feeling rushed for surgery.^47^

Nassiri et al., implemented a streamlined, patient-centered CI delivery model at a high-volume academic center, reducing the referral-to-surgery time from 136 to 24 days and minimized patient travel burden by integrating electronic medical records and coordinating same-day appointments and surgeries.^48^ Homans et al. piloted a multidisciplinary single-day cochlear implant selection protocol, reducing the selection process duration from 84 days to 1 day and hospital visits from 6 to 2. This protocol also led to a 27% decrease in professional time dedicated to CI selection.^49^

In another study, Homans et al. surveyed 37 CI users and 7 healthcare providers regarding the multidisciplinary single-day cochlear implant selection protocol, finding comparable satisfaction levels between this and standard protocols. The majority of CI users and practitioners preferred the single-day protocol, demonstrating its potential to streamline the CI selection process without compromising satisfaction.^50^ These studies collectively suggest that same-day consultation and surgery models can enhance patient convenience, and by minimizing travel time contribute to sustainability and support greener healthcare practices.

Combining multiple appointments into a single day can reduce travel time and thus combat climate change (SDG13), increase satisfaction and well-being among CI candidates (SDG3) and increase satisfaction among healthcare providers which promotes productive employment and decent work for all (SDG8).

## Summary and implications

While reaching for the ‘Green Cochlea’ and adopting sustainable practices in cochlear implantation offers promising benefits, several barriers may hinder the widespread implementation of these practices. Technological challenges include the development and integration of eco-friendly materials and alternative energy sources for CI components while ensuring they meet the high standards required for medical devices. Infrastructural challenges include the need for proper waste management systems capable of handling medical waste sustainably. Traditional healthcare settings are often not equipped with robust recycling programs or facilities to properly manage eco-friendly materials. Additionally, some hospitals may not have adequate systems for waste segregation, making the implementation of sustainable practices difficult. Healthcare-specific challenges might involve resistance to change and unwillingness to adopt new habits within the OR, or with how patient appointments are arranged. Lastly, economic challenges usually revolve around the higher upfront costs related to sustainable alternatives. Although these practices may result in long-term savings and environmental benefits, the initial financial burden can be a significant deterrent. This is particularly relevant in healthcare settings where budget constraints are already a significant concern.

**Fig. 2:**
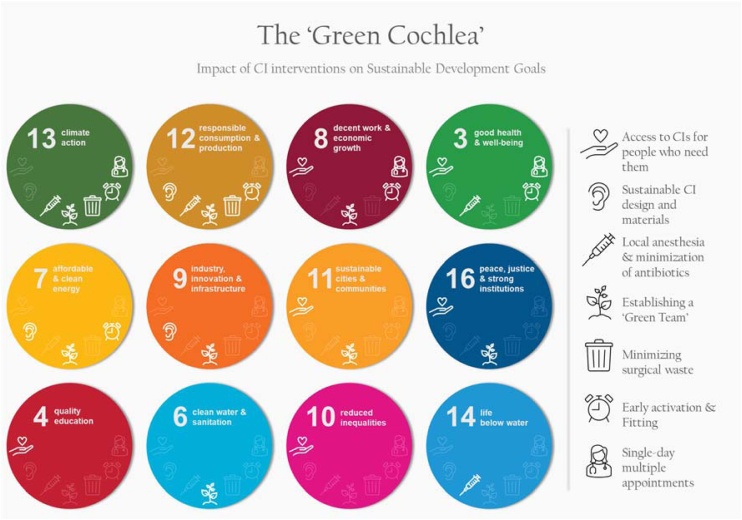
Impact of cochlear implantation interventions on the United Nations’ Sustainable Development Goals (SDGs). Only the SDGs that are affected are shown here (12/17), in order from the one affected by the most interventions (top left) to the one affected by the least interventions (bottom right). The icons inside the circles indicate which intervention affects each SDG and are explained in the legend on the right.

Addressing these challenges requires a multifaceted approach in various levels. Healthcare professionals can push policymakers for change in outdated practices such as the unnecessary printed material that accompanies each medical device. Within the healthcare organizations, creating a multidisciplinary and dedicated ‘Green Team’ is the first step towards detecting areas that need to be improved and adopting sustainable workplace practices. ‘Green Teams’ have been proven valuable in educating and inspiring employees around sustainability.^30^ Overall, the proposed interventions have the potential to impact and advance 12 out of the 17 United Nations’ SDGs (Fig. 2). By establishing the ‘Green Cochlea’ method and adopting more sustainable practices such as these the CI field has the opportunity to lead the way toward a greener healthcare.

## Conclusions

Prioritizing environmental sustainability in healthcare is an ethical imperative. Creating a sustainable ecosystem, such as the ‘Green Cochlea’, for CIs necessitates a close cooperation among clinicians, manufactures, and policymakers. Key findings from our study include the importance of adopting bio-based polymers and biodegradable plastics, transitioning to more sustainable energy solutions, implementing green anesthetic practices, and reducing surgical waste through better waste segregation and recycling practices. Promoting sustainable practices during postimplantation processes, such as early activation and minimizing patient visits, can further enhance both patient care and environmental impact. The transition to sustainable cochlear implantation will contribute to multiple Sustainable Development Goals (SDGs) set by the United Nations, promoting well-being, innovation, and climate action, thereby ensuring a healthier and more sustainable future for all.

## Justification for authorship exceeding seven

This study, The Green Cochlea, is a multidisciplinary collaboration that required expertise in various fields. Each author contributed significantly to the project’s design, execution, or analysis, reflecting the study’s broad scope and collaborative nature. All authors meet the established authorship criteria as defined by the International Committee of Medical Journal Editors (ICMJE).

## ORCID ID

Abdulrahman Hagr: 0000-0002-3561-6791

Farid Alzhrani: 0000-0002-0564-7204

Fida Almuhawas: 0000-0002-3354-8756

Yassin Abdelsamad: 0000-0001-8281-5825

Medhat Yousef: 0000-0003-2102-6704

Asma Alahmadi: 0000-0001-6603-1596

Christiana Kyvelidou: 0000-0001-6907-1542

Ilona Anderson: 0000-0001-7518-6661

## CRediT authorship contribution statement

A.H., F. Alz., and F. Alm. established the idea for this article; C.K, Y.A, and M.Y. performed the literature search and review; A.A., F. Alm., and I.A. wrote the first draft; A.H. and F. Alz. critically revised the work; all the authors commented on the edited version of the manuscript and approved the final one.

## Funding

No funding received to assist with the preparation of this manuscript.

## Declaration of competing interest

The authors declare no conflicts of interest.
